# Structure and molecular basis of spermatid elongation in the *Drosophila* testis

**DOI:** 10.1098/rsob.230136

**Published:** 2023-11-08

**Authors:** Qiuru Huang, Xia Chen, Hao Yu, Li Ji, Yi Shi, Xinmeng Cheng, Hao Chen, Jun Yu

**Affiliations:** ^1^ Institute of Reproductive Medicine, Medical School of Nantong University, Nantong University, Nantong, Jiangsu 226001, People's Republic of China; ^2^ Department of Obstetrics and Gynecology, Affiliated Hospital 2 of Nantong University, Nantong First People's Hospital, Nantong University, Nantong, Jiangsu 226001, People's Republic of China

**Keywords:** spermatid elongation, *Drosophila*, testis, axoneme assembly, individualization complexes, mitochondria

## Abstract

Spermatid elongation is a crucial event in the late stage of spermatogenesis in the *Drosophila* testis, eventually leading to the formation of mature sperm after meiosis. During spermatogenesis, significant structural and morphological changes take place in a cluster of post-meiotic germ cells, which are enclosed in a microenvironment surrounded by somatic cyst cells. Microtubule-based axoneme assembly, formation of individualization complexes and mitochondria maintenance are key processes involved in the differentiation of elongated spermatids. They provide important structural foundations for accessing male fertility. How these structures are constructed and maintained are basic questions in the *Drosophila* testis. Although the roles of several genes in different structures during the development of elongated spermatids have been elucidated, the relationships between them have not been widely studied. In addition, the genetic basis of spermatid elongation and the regulatory mechanisms involved have not been thoroughly investigated. In the present review, we focus on current knowledge with regard to spermatid axoneme assembly, individualization complex and mitochondria maintenance. We also touch upon promising directions for future research to unravel the underlying mechanisms of spermatid elongation in the *Drosophila* testis.

## Graphical abstract

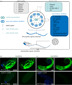

## 1. Introduction

*Drosophila* provides an excellent model for the characterization of functional genes contributing to male fertility [1,2]. More than 10% (approx. 1500 genes) of mutations in the *Drosophila* genome are associated with male fertility [3,4]. Spermatogenesis is a complex and highly regulated cell differentiation process in *Drosophila* and mammalian testes [5–7]. Starting with germline stem cells (GSCs), it leads to the formation of cysts having germ cells at the same developmental stage [8,9].

In *Drosophila*, spermatogenesis consists of three major stages: (1) pre-meiotic (GSCs and spermatogonia); (2) meiotic (spermatocytes); and (3) post-meiotic (round spermatids, elongated spermatids and sperm) [10,11]. At the apex of the *Drosophila* testis, 10–15 non-dividing hub cells are surrounded by GSCs and cyst stem cells (CySCs). Together, they form a specific testicular microenvironment called the ‘stem cell niche’, which plays an essential role in the maintenance and differentiation of stem cells [12]. Each GSC is enveloped by two CySCs and produces a daughter stem cell and a gonialblast (GB) by asymmetric cell division [13]. Each GB undergoes four rounds of transit-amplifying (TA) divisions, resulting in a cluster of 16 spermatogonial cells which enter the meiotic programme [6,8]. Ultimately, a cyst of germ cells is surrounded by two cyst cells connected by ring canals, leading to the production of 64 haploid spermatids within a cyst after the meiosis-I and meiosis-II divisions [8]. The post-meiotic spermatids undergo a series of ultrastructural changes, including axoneme assembly, mitochondrial morphogenesis and membrane remodelling, that eventually transform the round spermatids into mature motile sperm [7,14].

Previous research has already investigated in-depth the various key factors involved in spermatogenesis in the *Drosophila* testis, especially in the stem cell niche regulation and meiosis [15–21]. Recent studies have also thrown light on the various aspects of spermatid elongation in *Drosophila* [22–24]. The loss of function of several genes in *Drosophila* and mammals leads to similar testicular phenotypes, thereby indicating that their roles during spermatogenesis are conserved [3,25]. Moreover, several genes have been shown to play critical roles during spermatid deformation in the mammalian testis, including acrosome and flagellum formation, nuclear condensation, and cytoplasmic exclusion, finally transferring the round spermatids to the elongated spermatids [26–29]. Kang *et al.* [30] reported that the RNA binding protein FMR1 autosomal homologue 1 (FXR1) was required for spermatid development. It was shown to undergo liquid–liquid phase separation (LLPS), in order to link ribonucleoprotein granules with the translation machinery to promote translation initiation in mice [30]. Another recent study identified X-linked *terminal nucleotidyltransferase 5D* (TENT5D) as an oligoasthenoteratozoospermia (OAT)-related gene via whole-exome sequencing (WES) from Han Chinese men with OAT, and with the help of a gene-edited mouse model. TENT5D deficiency could cause defects in the spermatid maintenance by affecting the stability of mRNA during spermatogenesis [31]. These studies have highlighted the importance of spermatid differentiation in the final stage of spermatogenesis, and its crucial role in male fertility. Although several sources of information are available regarding the genetic regulation during spermatid differentiation, the structure and molecular basis of spermatid elongation in *Drosophila* remain open for further discussion. In this review, we discuss the major events and molecular basis for spermatid elongation, which may provide a theoretical basis for spermatogenesis and maintenance of male fertility.

## 2. Spermatid axoneme

### 2.1. Axoneme structure and microtubule arrangement

**Figure 1.**
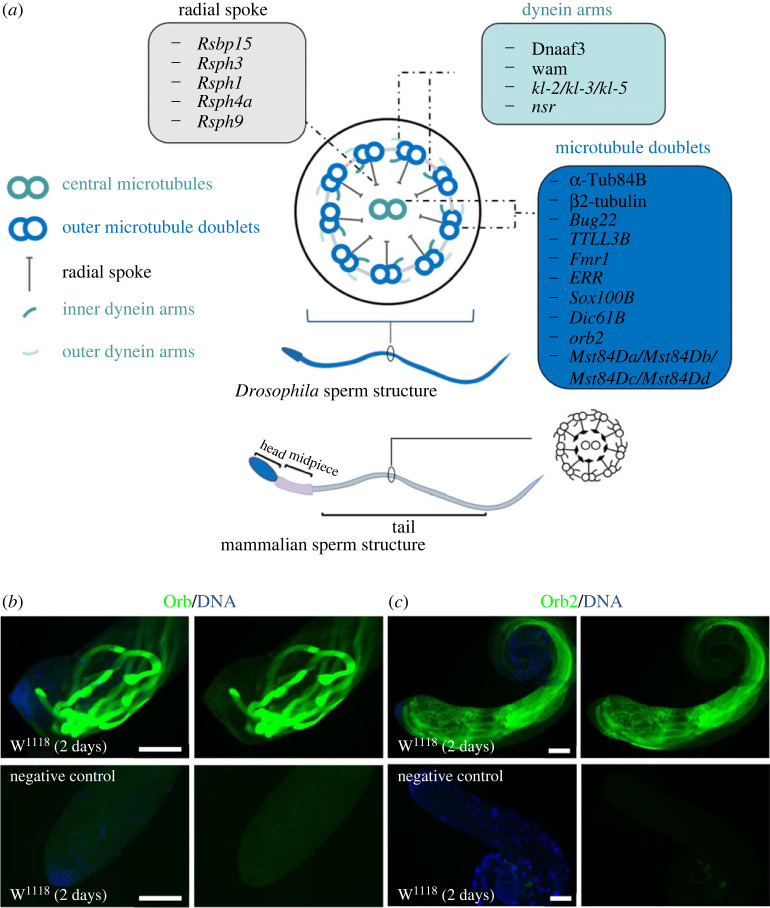
Schematic illustration of spermatid axoneme in *Drosophila*. (*a*) A schematic drawing illustrating the major structure of spermatid axoneme. Representative genes are shown for microtubule doublets, dynein arms and radial spoke. (*b,c*) Immunostaining of Orb (*b*) and Orb2 (*c*) for W^1118^ testes hatched at 2 days. DNA was stained with Hoechst. W^1118^ line is used as wild-type fly. Scale bar: 100 µm.

The spermatid axoneme consists of a microtubule-based cytoskeleton and is associated with sperm motility in *Drosophila* [7,32]. The axonemal structure of cilia and flagella is evolutionarily conserved in eukaryotes. It has a highly ordered microtubule-based ‘9 + 2’ arrangement, consisting of nine outer microtubule doublets surrounded by a central microtubule pair (figure 1*a*). Within the basic axoneme, the nine outer doublet microtubules are connected by nexin links and anchored by outer and inner dynein arms that mediate axoneme motility [4,33]. Outer doublet and central pair microtubules are connected by radial spokes as linkers and mechanochemical transducers, which transmit signals from the central pair apparatus to the microtubule doublets for local control of dynein activity [34–37].

### 2.2. Microtubule and tubulin modifications

Microtubules are assembled from tubulin heterodimers and are essential for maintaining cell shape, cell division and cell motility [38,39]. They are an important determinant of male fertility due to their role in the post-mitotic germ cells and some important tubulin subunits, such as α-tubulin at 84B (α-Tub84B) and testis-specific β2-tubulin, have been shown to function during spermatogenesis [39]. In particular, the cytoskeletal proteins that are specifically expressed in the testis play an important role in the microtubule structural components, which are directly involved in the maintenance of male fertility. Furthermore, post-translational modifications (PTMs) including polyglycylation, polyglutamylation and acetylation of tubulin proteins play a crucial role in the functioning of the microtubules, and are involved in the stability of centrioles and cilia [40]. The Basal body upregulated gene 22 (Bug22) has a highly conserved function during spermatid axoneme assembly via tubulin PTMs in both *Drosophila* and humans [40]. In addition, one of the glycosylase genes, *Tubulin tyrosine ligase-like 3B* (*TTLL3B*), is enriched in the testes of *Drosophila*. Daughterless-GAL4 (Da-Gal4) is a commonly used transgenic element that drives ubiquitous expression in *Drosophila* embryos, larvae and adults. The da-Gal4 driven loss of function of *TTLL3B* leads to axoneme disassembly and individualization defects in the *Drosophila* testis, indicating that polyglycylation plays an important role in the functioning of microtubules during spermatid individualization and axoneme assembly [41].

### 2.3. Axonemal dynein arm

A pair of dynein arms (outer and inner dynein arms) are an integral part of the axoneme structure and are located on both sides of the nine outer double-tubular microtubules [42]. Dynein motors consist of a large multi-subunit complex comprising heavy chains, intermediate chains and light chains. They regulate the ATP hydrolysis, motor activity and complex scaffold formation through these subunits [43]. Dynein axonemal assembly factor 3 (Dnaaf3) is one of the dynein assembly factors associated with primary ciliary dyskinesia [44]. The *Dnaaf3* mutants in *Drosophila* are male infertile with immotile sperm due the disruption of dynein arms and axoneme assembly in sperm bundles [45]. Dynein arms play an important role in the generation of the force needed for sperm motility [46]. A recent study has reported that wampa (wam) was an essential component of the outer dynein arm docking complex and required for attachment of the outer dynein arms to the axoneme of the sperm flagellum [47]. An increase in the proportion of abnormal individualization complexes, faulty localization of mitochondria, and malformation of the nucleus was observed in *wam* mutants during spermatogenesis, leading to the postulation that these mutants are male sterile [47]. The genes *male fertility factor kl2* (*kl-2*), *male fertility factor kl3* (*kl-3*), and *male fertility factor kl5* (*kl-5*) encode the outer dynein heavy chains of the sperm axoneme [48]. Functional studies indicate that mutations to *kl-3* and *kl-5* result in maintenance defects of the outer dynein arms in the axoneme of the sperm flagellum, while its effect on the spermatid individualization process is not clear [33,49]. Moreover, the *novel spermatogenesis regulator* (*nsr*) mutant results in male infertility due to its effect on the outer dynein arms in sperm axonemes and spermatid individualization [33]. The data suggest that *nsr* might control male fertility by regulating the Y-linked genes *kl-3* and *kl-5*.

### 2.4. Axoneme assembly

The flagellar wave form requires radial spokes and central tubules, and defects in these structures can lead to the bending of axonemes [4]. The central microtubule pair begins to nucleate a singlet microtubule within the basal body of a small cilium prior to meiotic divisions, and the second microtubule of the pair is assembled much later during flagella formation [50]. Fragile X syndrome (FraX) is the most common form of congenital mental retardation caused by the transcriptional silencing of the *Fragile X mental retardation 1* (*Fmr1*) gene. In *Fmr1* mutants, axonemes gradually lose the central microtubule pair [51]. The *Estrogen-related receptor* (*ERR*) is involved in the main pathway of cellular energy homeostasis, and *ERR* mutants die as larvae due to low levels of ATP and elevated levels of circulating sugars [52]. The *ERR* knockdown males are observed to have disrupted microtubule function and a significant reduction in the major mitochondrial derivatives [2]. *Sox100B* is an orthologue of vertebrate group E genes and is essential for testis development in *Drosophila* [53,54]. Interestingly, the nicked spermatid axoneme lacks the complete circular shape of the microtubular spoke after *Sox100B* knockdown [2]. It has been found that the radial spokes in *Dynein intermediate chain at 61B* (*Dic61B*) mutant testes were not uniformly organized, and more than two tubules could be visualized in the central pair, apparently due to the enlargement of some of the secondary fibres [4].

*Drosophila* oo18 RNA-binding protein (Orb) and Orb2 are two highly conserved RNA-binding proteins belonging to the cytoplasmic polyadenylation element binding (CPEB) protein family, which plays a critical role in mRNA transport and local translation [55]. Both Orb and Orb2 are highly enriched in elongated spermatids and regulate spermatogenesis [22,56]. Immunofluorescence staining in the *Drosophila* testis shows that Orb and Orb2 proteins have low levels of expression in mitotic spermatogonia and extremely high levels in elongated spermatids. The Orb2 protein is also present at an intermediate level of expression throughout the spermatocyte cytoplasm (figure 1*b*,*c*). Moreover, *orb2* mutants are male sterile, which can result from underlying defects in the assembly or localization of axonemal proteins, abnormal elongation of flagellar axonemes, insufficiently compact cyst bundles, and rough internal morphologies in elongated spermatid bundles [57]. By contrast, *orb* mutant males show weaker effects on male infertility than *orb2*, although Orb plays a critical role during oogenesis and *orb* mRNAs could be repressed by Orb2 [57]. These findings, in combination with the specific expression patterns of Orb during spermatid elongation, demonstrate that Orb has a certain role during spermatogenesis, which warrants further investigation.

Radial spoke proteins are functionally conserved in multiple species and show high similarities and identities with human homologues [58–60]. The radial spoke binding protein 15 (Rsbp15) is localized in the sperm flagellum and individualization complex (IC). The loss of *Rsbp15* results in male sterility, asynchronous IC, and defective axonemal structure in flagella. It resembles the phenotype of mutants of radial spoke-related genes, such as *Radial spoke head protein 3* (*Rsph3*), *Radial spoke head protein 1* (*Rsph1*), *Radial spoke head protein 4a* (*Rsph4a*), and *Radial spoke head protein 9* (*Rsph9*). The finding demonstrates that the structural integrity of the flagella is essential for the stability of IC [34]. Interestingly, Rsbp15 can interact with and stabilize Rsph3 through the DD_R_PKA superfamily domain [34].

*Male-specific RNA 84Da* (*Mst84Da*), *Male-specific RNA 84Db* (*Mst84Db*), *Male-specific RNA 84Dc* (*Mst84Dc*), *Male-specific RNA 84Dd* (*Mst84Dd*), and *Male-specific RNA 87F* (*Mst87F*) belong to a cluster of Mst(3)CGP gene family containing the repetitive Cys-Gly-Pro motif. These genes are male-specific and are exclusively transcribed in the primary spermatocytes in *Drosophila* testes [61]. *Mst84D* family genes are highly enriched in spermatids, as shown by single-cell RNA sequencing (scRNA-seq) [62,63], and can be used as potential markers for testicular spermatids in *Drosophila*. Importantly, the deletion of the *Mst84D* gene family results in various aberrations, including abnormal axoneme assembly, malformed nebenkern derivatives and reduction of motile sperms [61].

During spermatogenesis, the centrioles of the spermatids transform into basal bodies and form a template for the sperm flagellar axoneme [7,64]. Centrosomal protein 135 kDa (Cep135, also called Bld10) is an evolutionarily conserved centriole protein, which is a part of both the somatic centriole and the basal body. It plays a role in the proper formation of the axoneme and is associated with sperm motility, highlighting the importance of centriolar function in male fertility [7,65]. Moreover, *Drosophila* polo and Sak kinase (SAK), mitotic serine-threonine protein kinases, have been implicated in centrosome functions. Polo has been shown to be involved in centrosome maturation and mitotic progression while SAK is required for centriole identify and sperm axoneme formation [64].

## 3. Spermatid individualization

### 3.1. Individualization complex formation

**Figure 2.**
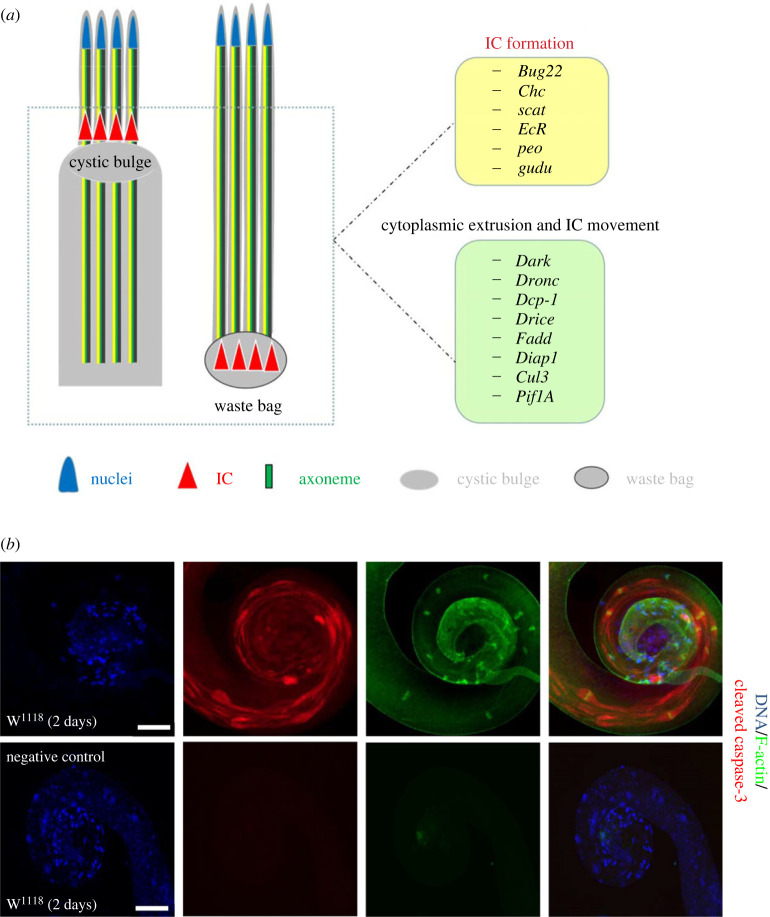
Schematic illustration of spermatid individualization in *Drosophila*. (*a*) A schematic drawing that illustrates the major structures in elongated spermatids. Critical genes for the formation and movement of IC and cytoplasmic extrusion are listed in the boxes. (*b*) Fluorescence images of the tail of the testis visualized by staining with F-actin (green) and cleaved Caspase-3 (red). DNA was stained with Hoechst. W^1118^ line is used as wild-type fly. Scale bar: 100 µm.

*Drosophila* provides an excellent system for visualizing the spermatogenic cyst, which could lead to a better understanding of sperm individualization [66]. During the late stage of spermatogenesis, fascicular elongated spermatids in clusters are separated from each other through the spermatid individualization process (figure 2*a*) [6,8]. The individualization process begins with an actin-based structure (also known as IC-investment cone) forming round the elongated nuclei [40]. Subsequently, the IC structure moves along the sperm tail, remodelling membrane and forming a voluminous structure called cystic bulge (CB) as a result of the continuous accumulation of extruded cellular material [40]. When the complex reaches the end of the spermatid tails, the CB turns into a waste bag (WB). It includes the excess cytoplasm and the minor mitochondrial derivatives, and is eventually shed into the lumen of the testis [8,40]. In the final step, individual sperms are coiled into the seminal vesicle and are ready for transfer to the female during copulation [8].

Spermatid individualization starts with the IC assembly, which consists of F-actin cones [40]. Individualization is a highly complicated process, and many factors are involved in the formation of IC. In the absence of *Bug22* in individualizing cysts, the IC is dispersed and lagged along the spermatid tails, while the CB and WB are smaller and contain less material [40]. The data suggest the occurrence of normal assembly of IC in the spermatid nuclei, but impair migration to individualized sperm tails in *Bug22* mutant testes. This is not a unique phenomenon, and has a counterpart wherein a *Clathrin heavy chain* (*Chc*) mutant fails to undergo individualization process and displays an altered IC distribution and morphology [66]. Other similar cases have been observed where *scattered* (*scat*), *Ecdysone receptor* (*EcR*) and *pendolino* (*peo*) mutants exhibit similar phenotypes of IC disruptions and nuclear scattering [66]. Another key factor, Gudu, shows high accumulation in adult testes. Its reduced expression leads to IC formation defects in spermatids, thereby causing a reduction in normal sperm production and resulting in low fertility [67].

### 3.2. Individualization complex movement

The successful movement of IC, in addition to its correct assembly, is an important factor affecting the process of individuation. There is relatively little information regarding the microtubule properties that enable the movement of actin cones along the axoneme. However, tubulin polyglycylation has been identified as a widely distributed PTM in ciliary microtubules and occurs in the early stage of the spermatid individualization [40,41]. In da-Gal4 driven *TTLL3B* RNAi testes, the spermatids are observed to be similar to the control in the early stage, with essentially normal axonemes as well as mitochondrial derivates. However, in the later stage of spermatid individualization, a large number of disorganized axoneme structures and reduced number of mitochondria are observed [41]. These studies demonstrate that polyglycylation is important for the axoneme assembly and plays a direct role in IC migration.

During spermatid individualization, cytoplasm and organelles are extruded between the sperm tails and pushed in front of the IC, forming a visible CB structure [68]. Interestingly, the movement of the actin cone away from the spermatid nuclei can be detected by co-staining of phalloidin (F-actin) and cleaved-Caspase-3 (figure 2*b*). Most of the cytoplasmic and cell organelles in CBs are degraded via a Caspase-3-mediated non-apoptotic pathway and deposited in WBs, which are eventually expelled [40,69,70].

*Drosophila Death-associated APAF1-related killer* (*Dark*) promotes the activation of the Death regulator Nedd2-like caspase (Dronc), which activates the downstream caspase proteins Death caspase-1 (Dcp-1) and Death related ICE-like caspase (Drice) [68]. Among the signalling pathways, the inhibition of *Dronc* function led to the formation of a large number of individualized spermatid cysts, which still contained large fingers with excess cytoplasm in individual spermatid units [68]. Fas-associated death domain (Fadd), the *Drosophila* homologue of mammalian FADD, is an adapter that mediates the recruitment of apical caspases to ligand-bound death receptors, which also requires the presence of its target caspase Death-related ced-3/Nedd2-like caspase (Dredd). Importantly, spermatids with *Fadd* mutant fail to individualize [68]. Furthermore, the *Death-associated inhibitor of apoptosis 1* (*Diap1*) encodes an E3 ubiquitin ligase, which is essential for preventing the inappropriate activation of caspase and apoptosis [70]. Moreover, loss of function of inhibitor of apoptosis protein (IAP) results in increased sensitivity of Fas-mediated cell death for germ cells [71]. Cullins are major components of another type of E3 ubiquitin ligase that serve as scaffolds for a catalytic module and a substrate recognition module [70]. Previous studies have shown that *cullin 3* (*Cul3*) was required for the activation of effector caspases in spermatids, thereby revealing the key role of ubiquitin signalling pathway in spermatid decellularization [70]. Moreover, PFTAIRE-interacting factor 1A (Pif1A) is highly enriched in spermatids and may localize to the front of actin cones. The loss of Pif1A leads to defects in actin cone movement, failed spermatid individualization and complete sterility [72,73].

Membrane remodelling is another major biological event that occurs along with IC movement [74,75]. During spermatid elongation, IC forms and progresses caudally along the spermatid cyst, remodelling the syncytial membrane to remove excess cytosol [76]. Plasma membrane remodelling defects can affect F-actin-based IC movement, therefore resulting in the individualization failure [77–79]. Several molecules have been elucidated to be associated with remodelling of the membrane during individualization. Jaguar (also known as myosin VI), a conserved myosin, is known to be involved in membrane dynamics. During spermatogenesis, dysfunctions in both dynamin and myosin VI can result in serious defects in the structure of actin, revealing the involvement of myosin VI and dynamin in regulating actin dynamics [80]. Niemann-Pick Type C (NPC) disease is an early childhood neurodegenerative disorder associated with mutations in *NPC intracellular cholesterol transporter 1* (*NPC1*) or *NPC intracellular cholesterol transporter 2* (*NPC2*). The *Drosophila Niemann-Pick type C-1a* (*Npc1a*) encodes a cholesterol-binding transmembrane protein, and its mutation leads to larval lethality, male infertility, and membrane remodelling defects during the individualization process [76]. Oxysterol-binding protein (Osbp) is involved in the non-vesicular transport, and distribution of intracellular sterols is also essential for spermatogenesis. Farinelli (Fan), a VAMP-related ER protein, regulates Osbp-mediated steroid transport and interacts with Osbp. Interestingly, both *fan* and *Osbp* mutants exhibit individualization defects in spermatids, which is similar to *NPC1* mutants, revealing the crucial role of cholesterol during individualization process [81]. Additionally, CDP-diacylglycerol synthase (CDS) is a highly important enzyme for lipid biosynthesis, and *Cds* mutations lead to multiple abnormalities in individualization, mitochondria and axonemal sheath [69].

## 4. Spermatid mitochondria

### 4.1. Mitochondrial functions in the *Drosophila* testis

**Figure 3.**
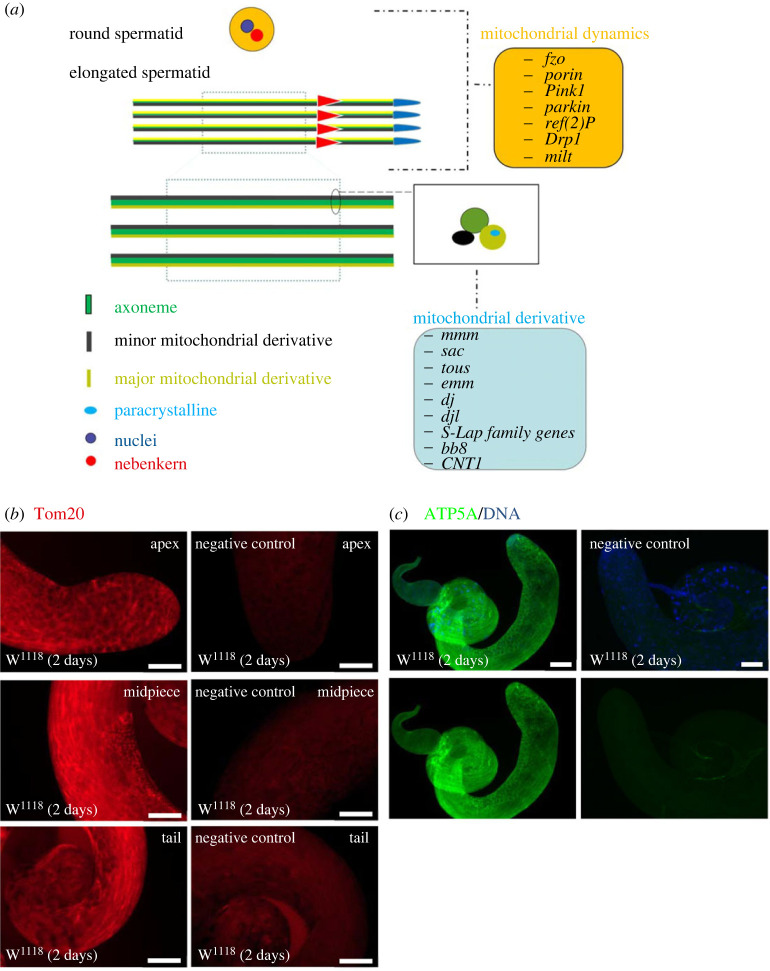
Schematic illustration of spermatid mitochondria in *Drosophila*. (*a*) A schematic drawing that illustrates the key factors for mitochondrial dynamics and mitochondrial derivatives. (*b*) Immunostaining of Tom20 (red) at the apex, midpiece and tail of W^1118^ testes to exhibit the distribution and morphology of mitochondria at different stages of spermatogenesis. At the apex of the testis, Tom20 labelled mitochondria display both aggregated and dispersed morphologies and are widely distributed in somatic and germline cells. At the midpiece of the testis, round spermatids can be clearly distinguished by onion-stage nebenkerns. At the tail of the testis, mitochondrial derivatives elongate along with spermatid axoneme, and can be labelled by multiple mitochondrial markers. (*c*) Immunostaining of ATP5A (green) for the whole mount of W^1118^ testes, which is similar to the result of Tom20 for mitochondrial distribution. DNA was stained with Hoechst. W^1118^ line is used as wild-type fly. Scale bar: 100 µm.

As an important functional organelle in both the somatic as well as germ cells, the mitochondrion plays a critical role in signal transduction, proliferation, differentiation, and cell death. This is in addition to its central role as the main source of energy and reactive oxygen species [82]. Mitochondrial morphology undergoes dynamic changes in shape and localization [83]. In mammals, the axoneme is surrounded by a sheath of ring-shaped mitochondria in post-meiotic spermatids [84]. By contrast to mammals, in the early post-meiotic *Drosophila* spermatids, mitochondria aggregate and fuse into two giant mitochondrial derivatives. These are interleaved by the onion stage to make a nebenkern [85]. Subsequently, the spermatids begin to elongate and the mitochondria detach from each other. The two mitochondrial derivatives can be seen elongating alongside the flagellar axoneme at the comet stage in the *Drosophila* testis (figure 3*a*) [8,86]. Several classic mitochondrial markers can be used to label mitochondria in *Drosophila* gonads [84,87]. In particular, our group uses Translocase of outer membrane 20 (Tom20) and ATP5A (also known as Bellwether (Blw)) to label mitochondria at different stages of germ cells in the *Drosophila* testis (figure 3*b*,*c*), which is helpful in understanding the regulatory mechanism of mitochondrial-related genes.

### 4.2. Mitochondrial dynamics

In *Drosophila*, the balance of mitochondrial dynamics between fusion and fission is essential for germ cell differentiation [88]. For example, *fuzzy onions* (*fzo*) was identified as a mitochondrial fusion gene encoding a transmembrane GTPase, and its dysfunction led to mitochondrial fusion defects and male infertility [89]. Porin is another important factor affecting mitochondrial fusion and fission, and its impaired expression adversely affects spermatid individualization, resulting in sterility [90]. *PTEN-induced putative kinase 1* (*pink1*) contains a mitochondrial-targeting motif and a serine/threonine kinase domain that are highly conserved in *Drosophila* as well as human [86,91]. Similar to the phenotype observed in *parkin* mutant testes, testicular spermatids lacking *pink1* display vacuolated onion-stage nebenkerns, and only a mitochondrial derivative is observed in the subsequent stage [86]. This finding indicates that *pink1* and *parkin* may play key roles in spermatids by reducing mitochondrial fission or increasing mitochondrial fusion. Overexpression of *parkin* rescues male sterility and mitochondrial morphology defects caused by the *pink1* mutation, thereby suggesting that *pink1* genetically interacts with *parkin* in *Drosophila* [92]. The mitochondrial protease subunit HtrA2 exhibits proapoptotic and cytoprotective properties, and its mutation leads to mild mitochondrial defects and male infertility. The *parkin* and *pink1* mutants share some phenotypic similarities, suggesting their involvement in maintaining mitochondrial integrity [93]. The gene *refractory to sigma P* (*ref(2)P*) is orthologous to the mammalian autophagy adapter gene *p62* and is a crucial downstream effector required for selective autophagy activation via multiple ubiquitinated proteins [94]. A previous study shows that *ref(2)P* is responsible for maintaining cellular mitochondria by promoting their aggregation and autophagic clearance [32]. Meanwhile, the loss of function of *ref(2)P* leads to several pathological phenotypes reminiscent of the *pink1* or *parkin* mutants, including mitochondrial abnormalities and mitochondrial DNA accumulation with heteroplasmic mutations, which may lead to defective locomotor activity in spermatid cells [32]. In addition, *ref(2)P* has been found to be involved in mitochondrial functions via the Pink1/Pakin pathway.

In addition, *Dynamin related protein 1* (*Drp1*) is well known to govern mitochondrial morphology in cells which play a central role in mitochondrial fission, such as neurons and lymph gland progenitors [95,96]. On the other hand, the *milton* (*milt*) gene encodes an adapter protein that connects mitochondria to kinesin, which is required for mitochondrial transport in *Drosophila* neurons [97]. Although both *Drp1* and *milton* are essential for mitochondrial dynamics, there are phenotypic differences in the mutant testes. The absence of *Drp1* leads to aberrant unfurling of the mitochondrial derivatives in early spermatids undergoing axonemal elongation, while the nebenkern is not strongly anchored to the nucleus and the mitochondrial derivatives do not properly elongate in *milt* mutant spermatids [97].

### 4.3. Mitochondrial derivative development

During spermatid elongation, the two mitochondrial derivatives behave differently: the major derivatives are filled with electron-dense paracrystalline array, while the minor derivative has a smaller volume and no paracrystalline accumulation [84,98]. Multiple factors can mediate the morphological abnormalities in the mitochondrial derivatives during spermatogenesis. Recently, Bauerly *et al*. identified three cilia-related genes, which include missing minor mitochondria (*mmm*), sterile affecting ciliogenesis (*sac*), and testes of unusual size (*tous*). The loss of function of these genes led to sperm flagellum immotility with the intact axoneme morphology [99]. As evidenced by mutations, mmm, sac and tous homozygotes resulted in the incorrect localization of mitochondria along the meiotic spindle, and plasma membrane defects due to cytokinetic errors [99]. The evidence indicates that *mmm*, *sac* and *tous* contribute to the maintenance of mitochondrial derivatives during or immediately after meiosis.

A previous study showed that emmenthal (emm) did not affect mitochondrial fusion or mitochondrial agglomeration in the meiosis and onion stage [100]. The study demonstrated that emm is required for the formation and maintenance of inner mitochondrial structure, starting from the stage of spermatocytes [100]. Furthermore, *don juan* (*dj*) and *don juan like* (*djl*) encode closely related proteins that localize in the cytoplasm of spermatocytes and the mitochondrial derivatives of elongated spermatids, revealing their functions associated with the mitochondria along with the flagella during spermatid elongation [101,102]. Paracrystalline array is thought to be associated with sperm tail elasticity and undulation, sperm-Leucylaminopeptidase (S-Lap) family proteins are identified from purified paracrystalline materials by mass spectrometry. Their mutants are male sterile, with defects in paracrystalline material accumulation and abnormal structure of the elongated major mitochondrial derivatives [103]. Vedelek *et al.* [84] showed that testis-specific expression of Big bubble 8 (Bb8) was highly enriched in the post-meiotic stages and localized in the mitochondria [84]. Interestingly, male flies lacking *bb8* became completely infertile and showed megamitochondria and the abnormal distribution of paracrystalline array in both mitochondrial derivatives during spermatid development in the *Drosophila* testis [84]. Another key factor is concentrative nucleoside transporter 1 (CNT1), which is required for the maintenance of major and minor mitochondrial derivatives in testis-specific manner. Loss of CNT1 expression leads to defects in spermatid maturation [104]. These findings suggest that mitochondrial dysfunction during spermatid elongation can cause damage by inducing multiple signalling pathways, the regulatory mechanisms of which need to be investigated further.

### 4.4. Mitochondrial nucleoid elimination

Mitochondria are specific organelles that contain their own genome, and most eukaryotes show a maternal inheritance of mitochondrial DNA (mtDNA) [105]. In *Drosophila*, mitochondrial nucleoid is normally eliminated by Endonuclease G (EndoG)-mediated process before spermatid individualization, while the mitochondria remain intact [106]. In fact, mtDNA removal from spermatid mitochondria is a special autophagy event that is evolutionarily conserved to prevent both the transmission of paternal mitochondrial DNA to the offspring and the establishment of heteroplasmy [107]. Mutation of mitochondria endonuclease *EndoG* results in retarded mitochondrial nucleoid elimination, whereas knockdown of *Drosophila tamas* (*tam*), the catalytic subunit of the mtDNA polymerase, has a much stronger effect on mitochondrial genome elimination [108]. The findings suggest that EndoG is dependent on Tam for the elimination of mitochondrial genomes.

## 5. Concluding remarks and future perspectives

In this review, we have conducted an in-depth analysis of spermatid elongation in *Drosophila*, which comprises a series of highly complicated and conserved biological events that have been historically underestimated. *Drosophila* has emerged as an excellent model system for the study of genetic functions and signalling pathways required for the late stage of spermatogenesis. Given the complexity of the process involved, it is clear that further studies are needed to elucidate the intrinsic molecular connections for structural organization and morphological changes during spermatid elongation.

When comparing expression of specific genes and the phenotypes resulting from their mutation, delays between the timing of expression and the phenotype are widely observed. For example, the RNA binding protein Maca is expressed specifically in spermatocyte nuclei, while the *maca* mutation exhibits IC defects during spermatid elongation, eventually leading to complete sterility [109]. On the other hand, many genes that are essential for the early stage of germ cells will also affect the late stage of spermatogenesis, particularly during axoneme assembly and spermatid individualization. Further investigations are needed to elucidate the exact mechanism. Our previous work has shown that changes in the expression levels of *CG6015* during spermatogonia transit-amplifying (TA) divisions inhibited the differentiation of germ cells. This led to the accumulation of GSC-like cell populations, and concomitantly reduced the number of elongated spermatid clusters in *Drosophila* testes [24]. Our recent study has also identified another key factor, eukaryotic disruption translation initiation factor 5 (eIF5), required for CySCs to promote germ cell differentiation via non-autonomous cellular effects. The disruption of eIF5 expression driven by tj-Gal4 in CySC lineage also led to severe defects in the F-actin labelled IC [56].

Although details of the interplay between spermatid axoneme, individualization and mitochondria remain largely unknown, it is observed that many of the genes participate in multiple regulatory roles. In addition to axonemal dynein arm, wam also plays a critical role in mitochondrial localization and nuclear head formation in spermatids [39]. Since lipid metabolism is an important factor in membrane curvature and function, spermatid elongation is involved in extensive membrane biosynthesis and remodelling [69,110]. The mutation of CDP-diacylglycerol synthase (Cds), which encodes an important enzyme in lipid biosynthetic pathway, leads to defects in both spermatid individualization and mitochondrial derivatives associated with the axonemes by affecting phosphatidylinositol synthase (dPIS) in *Drosophila* [69]. These findings suggest that many genes may play interlinked roles between microtubule based skeleton structure and mitochondrial derivatives, which may be elucidated in future studies.

Meanwhile, long noncoding RNAs (lncRNAs) are another cluster of cellular RNAs with functional roles in the regulation of many cellular developmental processes and diseases [111,112]. Although transcriptome profiling in multiple species has revealed the largest repertoire of lncRNAs in the testis [113–115], their functions and regulatory mechanisms in spermatogenesis remain largely unexplored. Using an optimized CRISPR/Cas9 system, Wen *et al.* [116] identified 33 out of 105 testis-specific lncRNAs required for male fertility and late development of spermatogenesis. The study provides a resource pool to further underline the specific regulatory mechanisms during spermatid elongation in the *Drosophila* testis [116].

In summary, this review explores the various structures involved in spermatid elongation and their genetic regulation in the *Drosophila* testis. Given the highly conserved nature of *Drosophila* and mammalian spermatogenesis, further study of spermatid elongation in *Drosophila* may provide a better understanding of the molecular basis and pathogenic phenotypes. This can, in turn, provide effective guidance for the clinical treatment of male infertility.

## Data Availability

The original contributions presented in the study are included in the article. Further inquiries can be directed to the corresponding authors.
